# Nontuberculous mycobacterial disease managed within UK primary care, 2006–2016

**DOI:** 10.1007/s10096-018-3315-6

**Published:** 2018-06-27

**Authors:** Eleanor L. Axson, Chloe I. Bloom, Jennifer K. Quint

**Affiliations:** Respiratory Epidemiology, Occupational Medicine and Public Health, National Heart and Lung Institute, Imperial College London, Emmanuel Kaye Building, Manresa Road, London, SW3 6LR UK

**Keywords:** Epidemiology, Primary care, NTM, COPD, Bronchiectasis

## Abstract

Previous UK studies investigating nontuberculous mycobacteria have been limited to reporting isolation from culture, not burden of disease. We assessed the burden of nontuberculous mycobacterial disease (NTMD) in UK primary care from 2006 to 2016. Using electronic healthcare records, we identified patients with NTMD using a strict definition including patients with guideline-directed treatment/monitoring. We described treatment regimens and incidence/prevalence in the general population and in patients with underlying chronic respiratory diseases. Incidence of primary care-managed NTMD in the general population decreased (2006 to 2016 rates per 100,000 person-years, 3.85 to 1.28). Average annual prevalence of NTMD in the general population was 6.38 per 100,000. Around 85% were taking antimycobacterial therapy; 53.2% were taking a guideline-recommended regimen. Incidence of NTMD in patients with respiratory disease decreased (2006 to 2016 rates per 100,000 person-years, 12.5 to 7.40). Average annual prevalence of NTMD in patients with respiratory disease was 27.7 per 100,000. This is the first UK study using nationally representative data to investigate the burden of NTMD managed within primary care. Incidence and prevalence of managed NTMD within primary care is gradually declining. Increasing complexity in the management of NTMD may be driving a shift in care to secondary settings.

**Electronic supplementary material:**

The online version of this article (10.1007/s10096-018-3315-6) contains supplementary material, which is available to authorized users.

## Introduction

Nontuberculous mycobacteria (NTM) are opportunistic bacterial pathogens [1–4]. Some populations are more susceptible to infection with NTM, particularly chronic respiratory disease (CRD) patients [3, 5–8]. The most common clinical manifestation of NTM disease (NTMD) is lung disease [2]. Previous studies have found that patients with chronic obstructive pulmonary disease (COPD), asthma, bronchiectasis, and cystic fibrosis (CF) experience higher NTMD burden than patients without those diseases [1, 7, 9–11].

National guidelines for the treatment of NTMD have changed over time and significant differences in recommended therapies were seen between the two major guidelines available during our study period [1, 12]. The new British Thoracic Society 2017 guidelines [2] have explicitly recommend specialist management of NTMD, unlike previous guidelines [1, 12].

Few studies have investigated the clinical burden of NTM infection in the UK; these small, secondary care-based studies found large variation in the proportion of isolates that resulted in actual clinical disease, but do not provide insight into NTMD at a national level [6, 13].

This is the first study to characterise NTMD patients, treatment regimens, and burden of NTMD, in the general population and in CRD patients, managed in UK primary care.

## Methods

### Data source

The Clinical Practice Research Datalink (CPRD) includes primary care records for 6.8% of the UK population, which are representative with respect to age, sex, BMI, and ethnicity [14–16].

### Study population

**Fig. 1:**
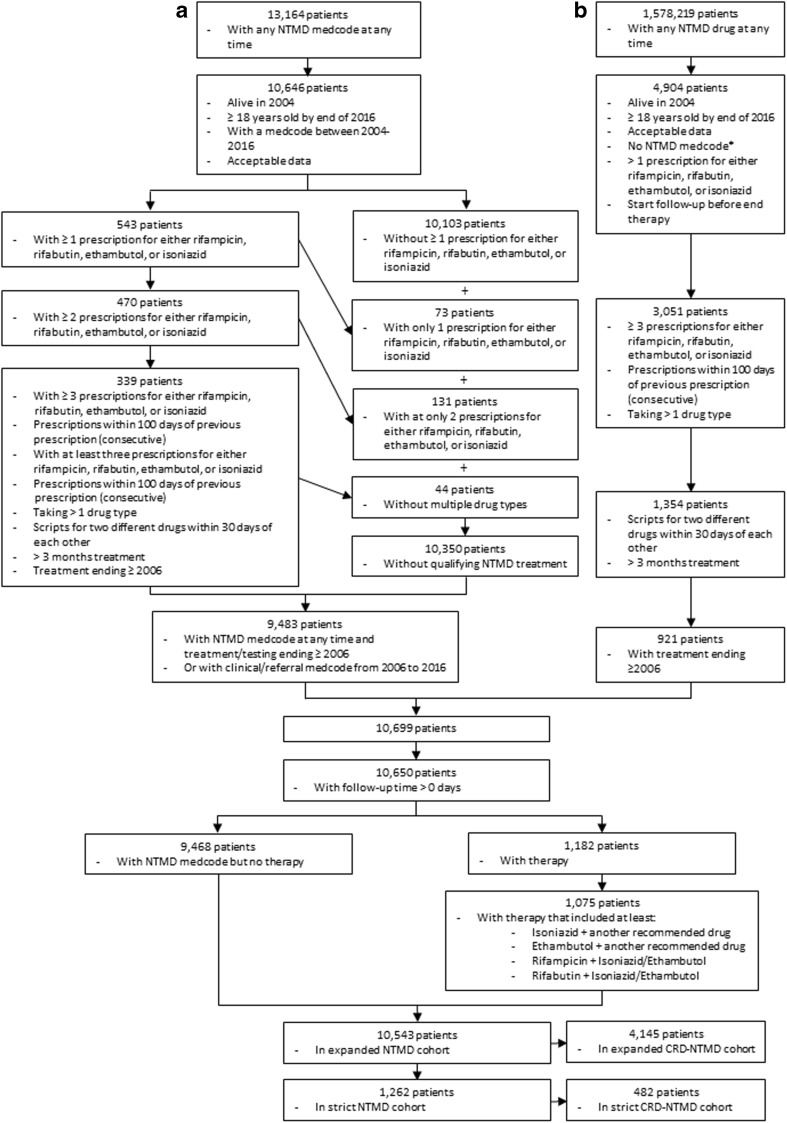
Mapping of patient inclusion. **a** Patients identified with NTMD medcodes. **b** Patients identified with prodcodes of drugs used in the treatment of NTMD. *Patients identified in the prodcode search who also had a medcode were mapped in the medcode scheme

We identified two adult cohorts (Fig. 1) using antimycobacterial prescription data and Read codes (the clinical terminology system used by CPRD) for NTMD between 01/01/2006 and 31/12/2016 (Sup. Tables 1–2). The cohorts were (1) a ‘strict cohort’, highly likely to have NTMD, and (2) an ‘expanded cohort’ including patients with possible NTMD. Comprehensive strict and expanded definitions of NTMD (Table 1) were used to ensure all likely and potential cases were identified. Briefly, the strict cohort comprised patients with evidence of appropriate primary care treatment/monitoring of NTMD, while the expanded cohort included all patients with any NTMD Read term during the study period, including a single sputum test. More details of specific inclusion and exclusion criteria are in the Supplementary Material. While we suspect the majority of our patients have NTMD located in the lungs, due to the nature of coding in CPRD, we cannot be certain of infection location. Thus, we refer to NTMD, not NTM lung disease.

**Table 1:** Strict and expanded definitions of nontuberculous mycobacterial disease (NTMD)

Strict NTMD definition	
1. Patients with 3+ NTM sputum tests coded, ≥ 3 months apart, over 2 years 2. Patients taking BTS00, ATS07, or BTS17 recommended multi-drug regimens for the treatment of NTMD, with 3+ consecutive prescriptions within 100 days of each other 3. Patients taking ≥ 2 of the 13 identified NTMD drugs with 3+ consecutive prescriptions within 100 days of each other, but must include any of the following: a. Isoniazid b. Ethambutol c. Rifampicin + isoniazid/ethambutol d. Rifabutin + isoniazid/ethambutol	
Expanded NTMD definition	
All of the above plus the following: 1. Patients with ≤ 2 NTM codes in the test file at any time without evidence of appropriate treatment 2. Patients with 1+ NTM codes in the clinical file at any time without evidence of appropriate treatment or repeated testing 3. Patients with 1+ NTM codes in the referral file at any time without evidence of appropriate treatment or repeated testing	

*BTS00*, British Thoracic Society 2000 recommendations [12]; *BTS17*, British Thoracic Society 2017 recommendations [2]; *ATS07*, American Thoracic Society/Infectious Disease Society of America 2007 recommendations [1]; *NTM*, nontuberculous mycobacteria

Sub-cohorts of patients with underlying CRD including COPD, asthma, bronchiectasis, CF, and interstitial lung disease (‘CRD cohorts’) were also analysed, as these populations have been identified as at greater risk for NTMD [3, 5–8]. Bronchiectasis, CF, and interstitial lung disease (ILD) were identified using specific Read terms (Sup. Table 2). Asthma [17] and COPD [18] were identified using validated algorithms. Patients could have more than one CRD diagnosis. This study included adults aged 18+ (COPD patients were aged ≥ 35 years), whose practice data was deemed to be ‘up-to-standard’ for research purposes by CPRD.

### Outcome and variables

The outcome was incident and prevalent NTMD, first episode only, managed in primary care stratified by age, sex, and calendar year of NTMD treatment/testing. Demographic variables are described in the Supplementary Material.

Recommended drug regimens were defined using the American Thoracic Society/Infectious Disease Society of American 2007 guidelines (ATS07) [1], the British Thoracic Society 2000 guidelines (BTS00) [12], and the British Thoracic Society 2017 guidelines (BTS17) [2]. Recommended regimens typically included at least one antimycobacterial drug in combination with a macrolide (Table 3).

### Calculating incidence and prevalence

Incidence of primary care managed NTMD was calculated by calendar year, sex, and age group. Period prevalence by calendar year, sex, and age group was calculated. Prevalence was not calculated for the expanded cohorts. These cohorts included patients identified using a single Read code on a single day, therefore contributing only 1 day to NTMD prevalent time so would not be a true representation of period prevalence. More details are in the Supplementary Material.

## Results

### The general cohort

The strict definition identified 1262 patients with NTMD; the expanded definition identified 10,543 (Table 2).

**Table 2:** Demographic and clinical characteristics for each cohort

	Strict NTMD general cohort *n* (%)	Expanded NTMD general cohort *n* (%)	Strict NTMD CRD cohort *n* (%)	Expanded NTMD CRD cohort *n* (%)
Number of patients	*n* = 1262	*n* = 10,543	*n* = 482	*n* = 4145
Female	601 (47.6)	5774 (54.8)	223 (46.3)	2132 (51.4)
Age at NTMD, years (mean, SD)	55.1 ± 18.5	56.0 ± 18.6	63.5 ± 14.8	62.6 ± 15.8
Males	55.5 ± 17.4	57.9 ± 18.0	62.9 ± 14.3	63.9 ± 15.5
Females	54.7 ± 19.6	54.4 ± 18.9	64.2 ± 15.3	61.4 ± 16.0
Age groups at NTMD				
18–34 years	234 (18.5)	1777 (16.9)	26 (5.39)	299 (7.21)
35–54 years	340 (26.9)	2924 (27.7)	80 (16.6)	811 (19.6)
55–74 years	482 (38.2)	3906 (37.1)	267 (55.4)	2027 (48.9)
75 years and over	206 (16.3)	1936 (18.4)	109 (22.6)	1008 (24.3)
Smoking status				
Never smoker/not recorded	592 (46.9)	5044 (47.8)	141 (29.3)	1461 (35.3)
Current smoker	332 (26.3)	2439 (23.1)	150 (31.1)	1176 (28.4)
Former smoker	338 (26.9)	3060 (29.0)	191 (39.6)	1508 (36.4)
Body mass index (kg/m^2^)	(*n* = 1156)	(*n* = 9725)	(*n* = 466)	(*n* = 4027)
Underweight (< 18.5)	126 (10.9)	468 (4.81)	65 (14.0)	243 (6.03)
Healthy weight (18.5–24.9)	549 (47.5)	3925 (40.4)	243 (52.2)	1597 (39.7)
Overweight (25.0–29.9)	322 (27.9)	3171 (32.6)	109 (23.4)	1287 (32.0)
Obese (> = 30)	159 (13.8)	2161 (22.2)	49 (10.5)	900 (22.4)
CRD comorbidities	*n* = 482 (38.2)	*n* = 4145 (39.3)		
Asthma	201 (15.9)	2250 (21.3)	201 (41.7)	2250 (54.3)
Bronchiectasis	157 (12.4)	1074 (10.2)	157 (35.6)	1074 (25.9)
Cystic fibrosis	10 (0.79)	35 (0.33)	10 (2.07)	35 (0.84)
COPD	309 (24.5)	2215 (21.0)	309 (64.1)	2215 (53.4)
Interstitial lung disease	30 (2.38)	220 (2.09)	30 (6.22)	220 (5.31)

Continuous data is reported as mean ± standard deviation. Categorical data is presented as count (percent). Percentages may not sum to 100 due to rounding. *NTMD*, nontuberculous mycobacterial disease; *COPD*, chronic obstructive pulmonary disease; *CRD*, chronic respiratory disease (asthma, bronchiectasis, cystic fibrosis, COPD, interstitial lung disease)

#### Characterisation of treatment regimen of strictly defined NTMD in the general cohort

Of the strict general cohort, 1075 (85.2%) patients were identified as taking an antimycobacterial therapy regimen, 74.5% for ≥ 12 months. Of those being treated for NTMD in primary care, 572 (53.2%) were taking a multi-drug regimen recommended by BTS00 [12], ATS07 [1], and/or BTS17 [2] (Table 3). A total of 503 patients (46.8%) were taking some other (non-recommended) combination of guideline-recommended drugs (Table 3). Patients without CRD were treated 88.2% of the time; those with CRD were treated 80.3% of the time. Treated patients without CRD were on a recommended regimen 41.7% of the time; 73.6% of treated patients with CRD were on a recommended regimen.

**Table 3:** Multi-drug regimens prescribed to patients with NTMD within primary care in the UK, 2004–2006

Multi-drug regimen for the treatment of NTMD	Patients *n* (% treated)	Regimen matches recommendations
Recommended regimens	572 (53.2)	
Rifampicin/rifabutin + ethambutol + isoniazid* (+ clarithromycin/azithromycin)	212 (19.7)	BTS00; ATS07; BTS17
Rifampicin/rifabutin + ethambutol	175 (16.3)	BTS00
Rifampicin/rifabutin + ethambutol + clarithromycin/azithromycin (+ amikacin)	155 (14.4)	BTS17; ATS07
Ethambutol + clarithromycin/azithromycin	19 (1.77)	ATS07**
Rifampicin/rifabutin + ethambutol + moxifloxacin (+ clarithromycin/azithromycin)	11 (1.02)	BTS17
Other/non-recommended combination	503 (46.8)	
Rifampicin/rifabutin + isoniazid* (+ clarithromycin/azithromycin)	355 (33.0)	
Isoniazid* + sulfamethoxazole (+ clarithromycin/azithromycin)	104 (9.67)	
Ethambutol + isoniazid*	13 (1.21)	
Rifampicin/rifabutin + isoniazid* + moxifloxacin	6 (0.56)	
Other***	25 (2.33)	
Total number of patients on therapy	1075 (100)	

*NTMD*, nontuberculous mycobacterial disease; *pts*, patients; *BTS00*, British Thoracic Society 2000 recommendations [12]; *BTS17*, British Thoracic Society 2017 recommendations [2]; *ATS07*, American Thoracic Society/Infectious Disease Society of America 2007 recommendations [1]. Drugs separated by a forward slash (/) mean that either drug may be used in the regimen. Drugs in parentheses are optional additions to the regimen. The counts of patients taking each regimen include patients taking either of the drugs separated by a forward slash and/or patients taking the regimen with or without the optional drugs in parentheses. *In all cases, isoniazid is recommended to be taken in combination with pyridoxine. **This combination was hesitantly recommended for only those with disease caused by *Mycobacterium avium* complex who had mild disease, medication intolerance, or disease suppression [1]. ***Other refers to some other combination of the 13 identified drugs, containing at least one of isoniazid, ethambutol, rifampicin + isoniazid/ethambutol, or rifabutin + isoniazid/ethambutol that individually had less than 5 patients

#### Incidence of strictly defined NTMD in the general cohort

**Fig. 2:**
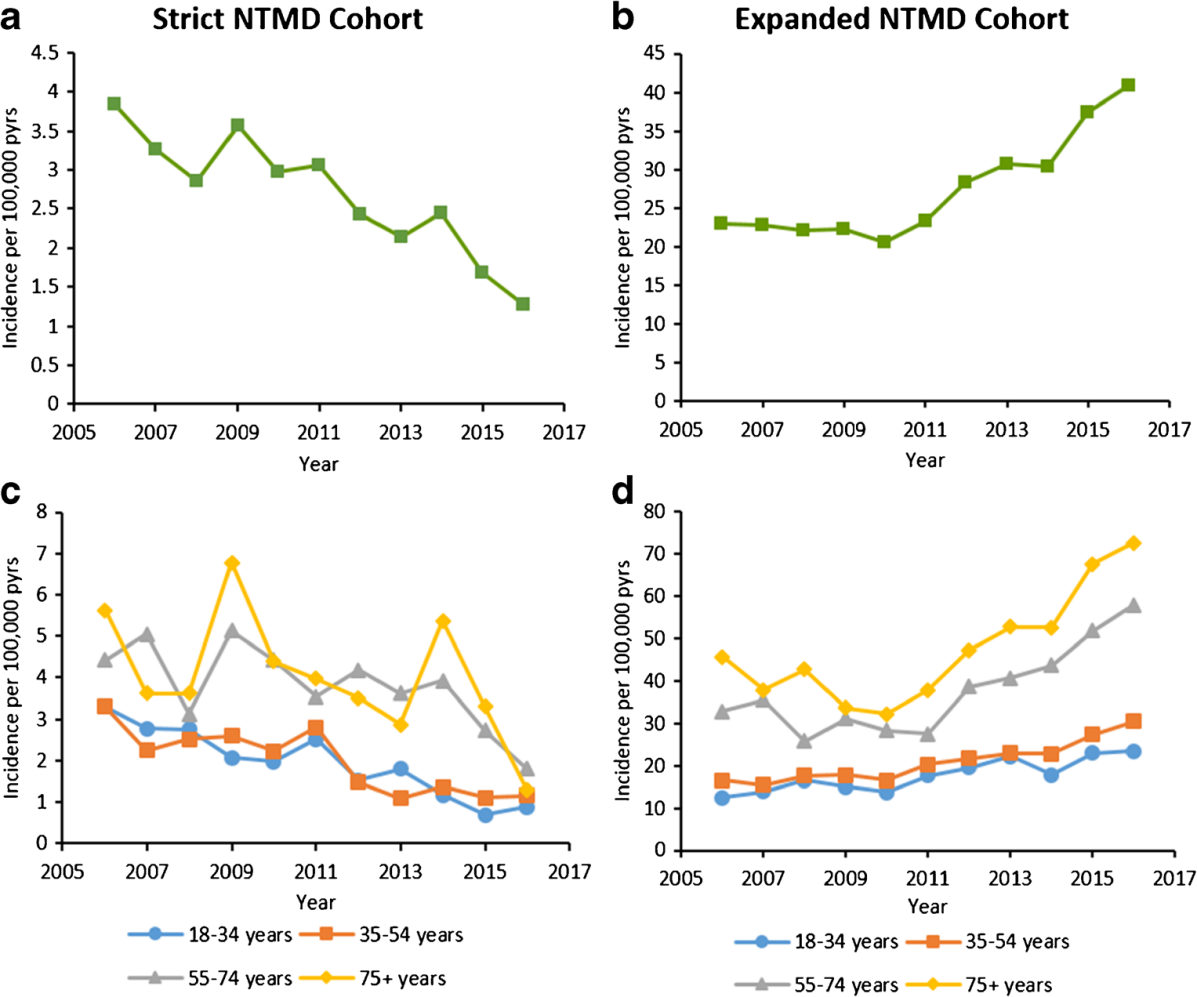
Annual incidence per 100,000 person-years of NTMD in UK primary care, 2006–2016. Presented overall for **a** strict cohort and **b** expanded cohort, and by age group for **c** strict cohort and **d** expanded cohort. NTMD, nontuberculous mycobacterial disease; pyrs, person-years

Incidence decreased from 2006 (3.85 per 100,000 person-years (pyrs)) to 2016 (1.28 per 100,000 pyrs) (Fig. 2; S3–4), for both sexes and within the three youngest age groups. There was no visible trend seen in the 75+ age group (Fig. 2; S3–4).

#### Prevalence of strictly defined NTMD in the general cohort

**Fig. 3:**
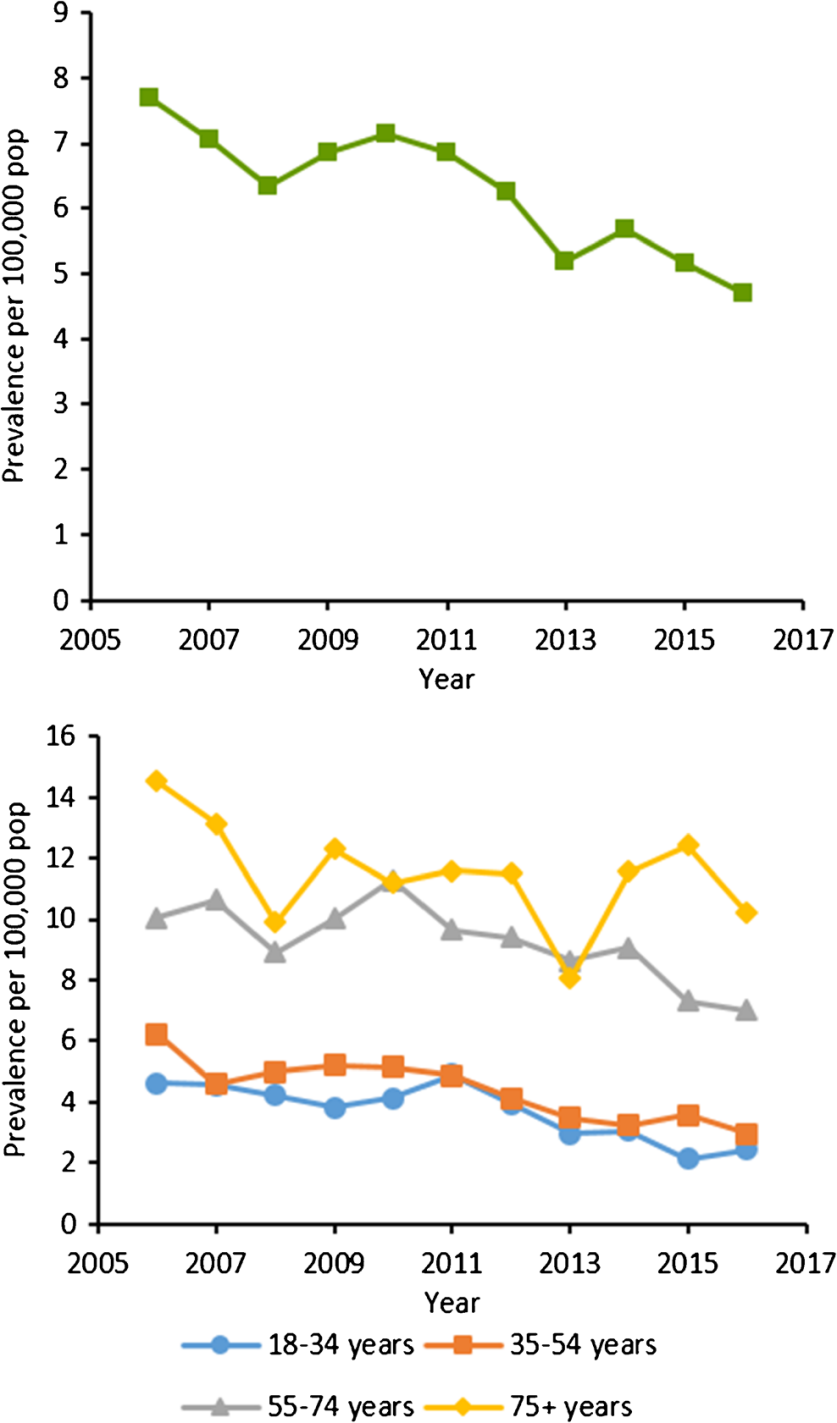
Annual prevalence per 100,000 population of NTMD in UK primary care, 2006–2016. Presented for our strict definition **a** overall and **b** by age-group for our strict NTMD definition. NTMD, nontuberculous mycobacterial disease; pop, population

Ten-year prevalence in UK primary care was 6.38 per 100,000. Prevalence of NTMD decreased from 2006 (7.68 per 100,000) to 2016 (4.70 per 100,000). Decreasing prevalence was seen in both sexes and all age groups (Fig. 3; S6–7).

#### Incidence of expanded definition of NTMD in the general cohort

Using our expanded definition of NTMD, incidence increased from 2006 (22.9 per 100,000 pyrs) to 2016 (40.9 per 100,000 pyrs) (Fig. 2; S3–4). Incidence increased for both sexes and in all age groups (Fig. 2; S3–4).

### The CRD cohort

Using our strict definition, we identified 482 NTMD patients with CRD; the expanded definition identified 4145 patients (Table 2).

#### Incidence of strictly defined NTMD in the CRD cohort

**Fig. 4:**
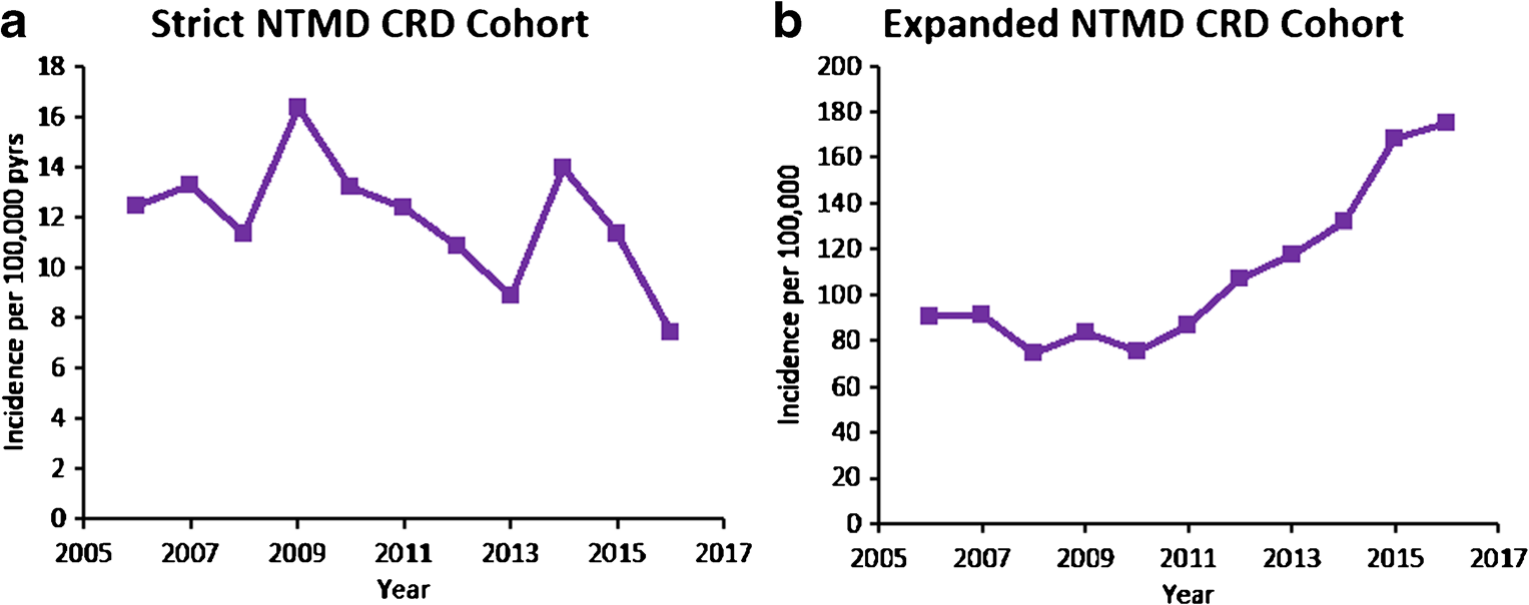
Annual incidence per 100,000 person-years of NTMD in patients with underlying respiratory disease in UK primary care, 2006–2016. Presented overall for **a** strict cohort and **b** expanded cohort. CRD, chronic respiratory disease; NTMD, nontuberculous mycobacterial disease; pyrs, person-years

Incidence of NTMD in our strict underlying respiratory disease cohort showed an overall downward trend from 12.5 per 100,000 pyrs in 2006 to 7.40 per 100,000 pyrs in 2016 (Fig. 4; S5).

#### Prevalence of strictly defined NTMD in the CRD cohort

**Fig. 5:**
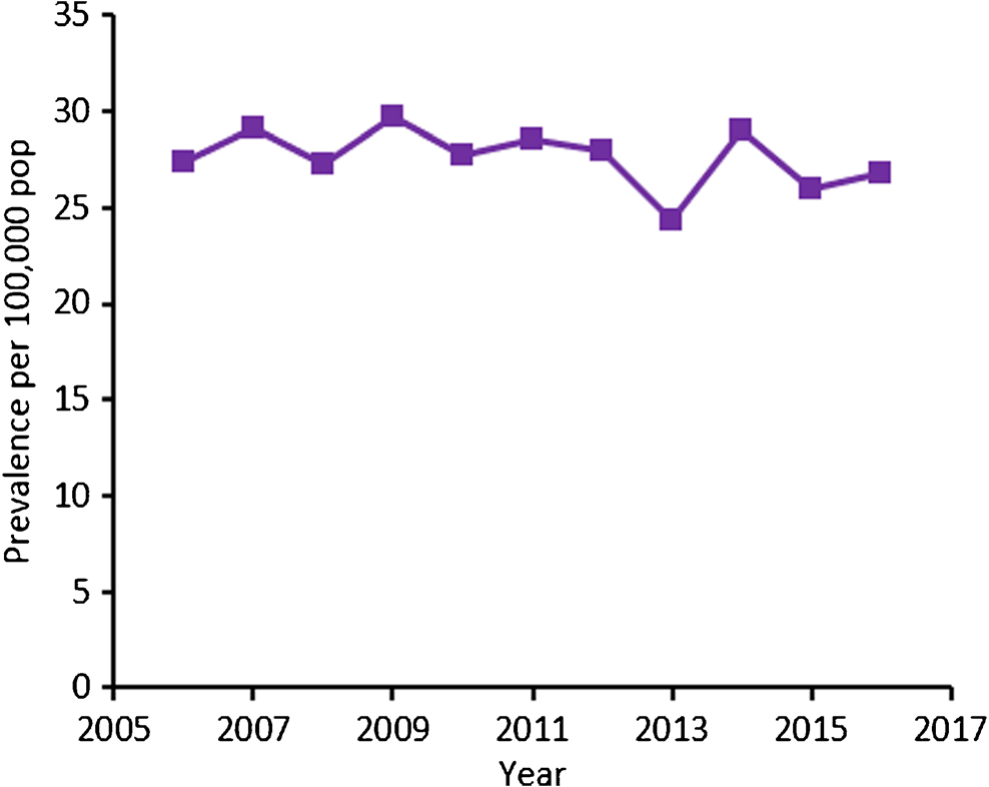
Annual prevalence per 100,000 population of NTMD in patients with underlying respiratory disease in UK primary care, 2006–2016. Presented for our strict NTMD definition in patients with underlying respiratory disease. NTMD, nontuberculous mycobacterial disease; pop, population

Ten-year prevalence of NTMD in patients with underlying CRD in UK primary care was 27.7 per 100,000. Prevalence of NTMD remained constant from 2006 to 2016 (Fig. 5; S8).

#### Incidence of expanded definition NTMD in the CRD cohort

Incidence of NTMD in our expanded underlying respiratory disease cohort increased from 2006 (90.6 per 100,000 pyrs) to 2016 (175.2 per 100,000 pyrs) (Fig. 3; S5).

## Discussion

This is the first national-scale study to look at the incidence and prevalence of NTMD managed within UK primary care, and investigate this within a CRD population.

Prevalence of NTMD was slightly higher in males than females in both strict cohorts, in keeping with most other studies [3, 7, 13, 19–22]. The average ages of our general cohorts are similar to other European populations [11, 21, 22]; however, there was an unusually large proportion of young persons in our strict general cohort. This may be indicative of young, healthier, milder disease being managed in primary care, whereas older, frailer, more severe disease is managed in secondary care. As in previous studies, a notable proportion of patients were underweight [23].

Just under 40% of both general NTMD cohorts had a CRD. COPD was the most common in the strict cohort, followed by asthma, following previous studies [11, 13, 21, 22]. However, bronchiectasis was slightly lower in our cohorts than seen previously and could be due to our primary care population as NTMD-bronchiectasis patients may be more likely seen in secondary care [11, 13, 21, 22]. ILD was fourth in both cohorts and we found no previously published studies to explicitly estimate the amount of comorbid NTMD and ILD. We found, as expected, a very low proportion of CF patients with NTMD, most likely as CF is uncommon, our adult-only cohort, and CF patients being treated predominantly by specialists.

In our study, 14.8% of patients in our general strict cohort had no evidence of NTMD treatment, less than a recent German study [24]. Treatment of NTMD in UK primary care appeared to follow guidelines available during our study period only half of the time [1, 12]; however, when considering only the ATS07 [1], the most commonly used worldwide [25], treatments were concordant only 36% of the time. Many treated patients meeting guidelines (16%) were taking rifampicin/rifabutin and ethambutol, a combination recommended by BTS00 [2], but not subsequent guidelines [1, 2]. Patients without a history of CRD were more often treated than those with CRD; however, patients with CRD were more likely to be on recommended NTMD treatment regimens than patients without CRD. This suggests that patients with CRD may be more likely to be treated in secondary care and/or treated in consultation with a specialist than those without CRD.

Incidence in our strictly defined NTMD general cohort decreased over time, running counter to evidence that isolation of NTM from clinical samples and NTMD is increasing globally [11, 26–32]. Specifically, in the UK, excluding Scotland, isolation from clinical samples has grown almost tenfold [3, 20]. Scotland and Denmark [19, 33] have seen stable NTM occurrence and nowhere has seen decreases. Decreasing NTMD in our strictly defined general cohort, requiring evidence of appropriate therapy or repeated testing, could suggest that the management of NTMD patients in the UK is shifting towards secondary care. This is supported by findings observing steep increases in NTM isolation in UK secondary care [3, 34]. This may be due to changing risk factors, particularly increased use of immunosuppressant drugs and inhaled corticosteroids associated with acquisition of NTM infection [35, 36] and requiring more complicated management. For example, guidelines recommend immunosuppressant drugs for the treatment of rheumatoid arthritis [37] and inflammatory bowel syndrome [38], and appear in clinical trials for treatment of asthma [39] with some recommended for use [40]. Increasing complexity of NTMD management is reflected in the most recent BTS guidelines, which explicitly recommend specialist management of NTMD [2].

As in previous studies [19], incidence of NTMD increased with increasing age in all our cohorts. For both definitions of NTMD, incidence was higher in patients with CRD than in the general population; this could suggest that patients with a history of CRD are more likely to suffer from, or be monitored for, NTM infection than the wider population.

Contrarily, incidences in our expanded definition cohorts increased over time. Our expanded definition of NTMD included all eligible CPRD patients with any NTMD code, including a single test, suggesting general practitioners (GPs) may be testing for NTMD more. This study provides a baseline for understanding the impact of the BTS17 guidelines, which explicitly recommend management by NTMD specialists; however, discrepancies between our strict and expanded cohorts may indicate that NTMD in the UK may already be principally managed in secondary care. Additionally, we found 14.8% of our patients with likely NTMD had no evidence of treatment within primary care; indicating that management coding in primary care is incomplete, management of NTMD is shared by primary and secondary care, and/or identification of NTMD occurs in primary care but is managed solely in secondary care.

### Limitations

This study only used primary care data to identify patients with NTMD, limiting generalisability to the wider UK population. Microbiological data was not available, limiting our accuracy in identifying NTMD cases. Several steps were taken to limit misclassification of patients, as described in the methods and limitations Supplementary Material. We used an expanded definition to try to capture all NTMD, including patients who were only partly managed within primary care, but undoubtedly the use of this definition included patients who were only investigated, but not diagnosed.

We are limited to reporting NTMD managed in primary care and cannot generalise to the whole UK population. Unfortunately, available secondary care data was not granular enough to pick up TB clinics, where NTMD patients are most likely managed. Without detailed secondary care and microbiology data, it cannot be known whether a shift in NTMD management from primary to secondary care has occurred, although other findings support our hypothesis [3, 34].

## Conclusions

This is the first nationally representative UK study to investigate the clinical burden of NTMD managed within primary care. We have shown that, using a strict NTMD definition referring to treated or monitored patients, incidence and prevalence within primary care is gradually declining; prevalence remained steady in patients with CRD. Only ~ 50% of primary care NTMD patients appeared to be treated according to guidelines. Increasing complexity in NTMD management may be driving a shift towards secondary settings, with an increased awareness for identification in primary care.

## Electronic supplementary material

ESM 1 (DOCX 66 kb)
