# Supplementary material for: Nontuberculous mycobacterial disease managed within UK primary care, 2006–2016
Source: Eur J Clin Microbiol Infect Dis. 2018 Jun 27;37(9):1795–803. doi: 10.1007/s10096-018-3315-6 (PMC6133048; doi:10.1007/s10096-018-3315-6)

**Supplementary Material**

**Study Population**

Due to the clinical difficulty in diagnosis and possible misclassification of cases (for example, using tuberculosis (TB) Read codes as an umbrella for any mycobacterial disease in the lung), we used this two-layer approach for case finding and report results for both cohorts. We excluded patients taking rifampicin and isoniazid for ≤3 months and patients taking lone isoniazid as these are the National Institute for Health and Care Excellence (NICE) guidelines for the treatment of latent TB [1]. We assumed active TB was not treated in primary care as general practitioners adhere to National Institute for Health and Care Excellence (NICE) guidelines, which recommend that latent and active TB patients be referred to a TB specialist [1,2].

**Outcomes and Variables**

Age at NTMD referred to the age at which treatment or regular testing began, or first date of clinical/referral medical code. Smoking history was identified using Read codes [21]. Body mass index (BMI) was measured as kg/m^2^ (underweight <18.5, healthy weight = 18.5-25, overweight = 25-30, obese ≥30). Age groups were 20-year age bands (18-34yrs, 35-54yrs, 55-74yrs, and 75+yrs).

**Calculating Incidence**

Incidence of managed NTMD was calculated by calendar year, sex, and age group by dividing the number of new NTMD cases by the person-time at risk in the general population or the adult respiratory disease population, as appropriate. For incidence calculations, patients with prevalent NTMD at the start of the study were excluded.

**Calculating Prevalence**

A patient was considered to have prevalent NTMD from their first date of NTMD treatment/testing until their last consecutive prescription or testing date. Period prevalence by calendar year, sex, and age group was calculated by dividing the number of NTMD cases starting or continuing treatment/testing during each given year by the total adult population, or adult respiratory disease population, at the midpoint of that year. Prevalence was not calculated for the cohorts identified using the expanded definition. These cohorts included patients identified using a single Read code on a single day, therefore contributing only 1 day to NTMD prevalent time so would not be a true representation of period prevalence.

**Limitations**

We did not have linked microbiological data therefore misclassification of NTMD was possible. We took a number of steps to ensure as accurate a classification as possible in our strict cohort. Firstly, we identified patients taking multi-drug combinations that are guideline-recommended for the treatment of NTMD, as well as patients on other combinations not explicitly recommended, but consisting of recommended drugs [3-5]. Secondly, we excluded all patients taking any multi-drug regimen for <3 months. Thirdly, we excluded patients taking rifampicin in the absence of ethambutol or isoniazid; rifampicin can be used to treat other infections [6] and recommended regimens for NTMD have rifampicin paired with at least one other antimycobacterial [3-5]. Lastly, we excluded patients with <3 sputum samples sent for NTM as three is the guideline-recommended number of samples to be taken, with at least two of these being positive for diagnosis of NTMD [4,5]. It is possible that a small number of included patients were being treated with antimycobacterial drugs for other reasons; but, importantly, treatment for latent or active TB is unlikely in primary care [2]. We used an expanded definition to try to capture all NTMD, including patients who were only partly managed within primary care, but undoubtedly the use of this definition included patients who were only investigated for NTMD and did not eventually receive a diagnosis of clinical disease.

We cannot report whether patients were taking the recommended multi-drug regimen for the specific NTM species causing their disease. We also could not distinguish between pulmonary and non-pulmonary NTMD, although it is likely most patients had pulmonary disease, particularly those in the CRD cohorts. Disseminated NTMD mainly occurs in HIV patients and there were few in our cohorts; HIV in the UK is managed outside of primary care and GPs may not be aware of the HIV status of their patients, so the exact proportion of likely cases of disseminated disease in our cohorts is unknown.

We are limited to reporting NTMD that was treated or managed in primary care and thus cannot generalise to the whole UK population, including secondary care, though our sensitivity analysis did not find that using inpatient HES data added information. Unfortunately, HES outpatient data is not granular enough to pick up TB clinic data, where it is most likely that NTMD patients are managed. Without detailed secondary care studies and linked microbiology data, it cannot be known whether there has been an increase in NTMD management in secondary care that parallels the decrease in primary care that we see here, although other findings support our hypothesis [7,8].

**References**

1. NICE (2016) Tuberculosis. nice.org.uk/guidance/ng33. Accessed Oct 2017

2. Atchison C, Zenner D, Barnett L, Pareek M (2015) Treating latent TB in primary care: a survey of enablers and barriers among UK General Practitioners. BMC Infect Dis 15:331. doi:10.1186/s12879-015-1091-9

3. Campbell I, Drobniewski F, Novelli V, Ormerod P, Pozniak A, Society SotJTCotBT (2000) Management of opportunist mycobacterial infections: Joint Tuberculosis Committee guidelines 1999. Thorax 55:210-218

4. Griffith DE, Aksamit T, Brown-Elliott BA, Catanzaro A, Daley C, Gordin F, Holland SM, Horsburgh R, Huitt G, Iademarco MF, Iseman M, Olivier K, Ruoss S, von Reyn CF, Wallace RJ, Jr., Winthrop K, Subcommittee ATSMD, American Thoracic S, Infectious Disease Society of A (2007) An official ATS/IDSA statement: diagnosis, treatment, and prevention of nontuberculous mycobacterial diseases. Am J Respir Crit Care Med 175 (4):367-416. doi:10.1164/rccm.200604-571ST

5. Haworth CS, Banks J, Capstick T, Fisher AJ, Gorsuch T, Laurenson IF, Leitch A, Loebinger MR, Milburn HJ, Nightingale M, Ormerod P, Shingadia D, Smith S, Whitehead N, Wilson R, Floto RA, Group BNGD (2017) British Thoracic Society Guidelines for the Management of Non-Tuberculous Mycobacterial Pulmonary Disease (NTM-PD). Thorax 72 (Supplement 2)

6. (BNF) BNF (2017) Rifampicin. National Institute for Health and Care Excellence (NICE). <https://bnf.nice.org.uk/drug/rifampicin.html>. Accessed Oct 2017

7. Cowman S, Burns K, Benson S, Wilson R, Loebinger MR (2016) The antimicrobial susceptibility of non-tuberculous mycobacteria. J Infect 72 (3):324-331. doi:10.1016/j.jinf.2015.12.007

8. Moore J, Kruijshaar M, Ormerod LP, Drobniewski F, Abubakar I (2010) Increasing reports of non-tuberculous mycobacteria in England, Wales and Northern Ireland, 1995-2006. BMC Public Health 10:612

| **Drug** | **CPRD Prodcodes** |
| --- | --- |
| Amikacin | 21498, 26770, 28915 |
| Azithromycin | 743, 5057, 5335, 14514, 33888, 40218, 43400, 46695, 49530, 53850, 58206, 58426, 60814, 61810, 65486, 66034, 65855, 4165, 5116, 52029, 60382, 62785 |
| Clarithromycin | 5662, 11433, 15290, 331, 537, 681, 765, 2719, 3736, 5357, 6121, 6623, 6803, 9583, 10326, 13323, 14816, 17645, 26059, 28289, 28349, 31689, 31690, 34394, 34533, 34608, 34650, 34811, 34974, 38163, 38997, 39010, 40784, 41453, 45591, 45795, 46488, 47582, 48023, 48163, 49939, 50946, 51154, 51426, 51831, 52158, 52411, 52719, 53086, 53109, 53144, 53153, 53168, 53179, 53398, 53688, 53703, 53715, 53776, 53875, 54208, 54241, 54269, 54472, 54529, 54882, 54897, 54953, 55148, 55428, 57267, 57660, 58037, 58175, 58902, 60805, 61001, 61830, 62897, 63033, 63236, 65259, 66092, 67560, 68723, 68943, 6497 |
| Ethambutol | 5101, 5102, 22266, 23246, 32316, 34036, 34037 |
| Ethambutol + Isoniazid | 8295, 8603, 23652, 25297, 27474, 28061, 29231, 31651 |
| Isoniazid | 2797, 6535, 6875, 27143, 41640, 41642, 49717, 51944, 52346, 63347, 65915, 66987, 32964, 26877, 21667, 15536, 29549, 12163, 29544, 15541, 17862, 17860, 32468, 15542, 28319, 22099, 8604, 15543, 21082, 25954, 56404, 30632, 27087, 22100, 356 |
| Linezolid | 29295, 29541, 21195, 23407 |
| Moxifloxacin | 6306, 25127, 43123, 47995 |
| Rifabutin | 5825, 18276 |
| Rifampicin | 240, 1345, 1347, 1531, 2798, 11226, 13908, 16639, 21367, 25277, 32407, 34130, 34653, 36357, 38661, 42496, 46917, 63931 |
| Rifampicin + Isoniazid | 3403, 4522, 8613, 9062, 12329, 12437, 16802, 26776, 39020, 39194, 57602, 58343 |
| Sulfamethoxazole/trimethoprim | 131, 287, 303, 1634, 3660, 8286, 8741, 9100, 10308, 10318, 15988, 16620, 20126, 20368, 20920, 25269, 25908, 27418, 27445, 28004, 30614, 31463, 31905, 33794, 33987, 41991, 44241, 45757, 46663, 60216, 60448, 67613, 109, 135, 606, 1199, 1467, 1604, 2460, 2658, 7420, 7421, 8561, 10745, 21809, 22991, 27921, 29907, 30201, 31477, 31484, 34727, 38090, 41579, 41967, 41978, 42517, 43262, 44075, 44286, 52198, 54914, 58282, 59444, 63733, 65343, 68027, 68101, 68726, 68826, 68990 |

**Supplementary Table 1: Code list for medications used to treat NTMD in UK primary care as identified using the BTS00, ATS07, and/or BTS17 treatment guidelines.** NTMD = nontuberculous mycobacterial disease. CPRD = Clinical Practice Research Datalink.

| **Disease** | **CPRD Medcodes** |
| --- | --- |
| Nontuberculous Mycobacterial Disease | 7202, 14557, 24425, 28623, 32223, 37006, 38141, 38271, 38558, 43568, 47652, 59544, 65529, 67609, 73936, 90273, 93266, 96623, 96793, 109496 |
| Asthma | 78, 81, 185, 232, 233, 1555, 2290, 3018, 3366, 3458, 3665, 4442, 4606, 4892, 5267, 5627, 5798, 5867, 6707, 7058, 7146, 7191, 7378, 7416, 7731, 8335, 8355, 9018, 9552, 9663, 10043, 10274, 10487, 11370, 12987, 13064, 13065, 13175, 13176, 14777, 15248, 16070, 16667, 16785, 18223, 18224, 18323, 19167, 19519, 19520, 19539, 20860, 20886, 21232, 22752, 24479, 24506, 24884, 25181, 25791, 26501, 26503, 26504, 26506, 26861, 27926, 29325, 30458, 30815, 31167, 31225, 38143, 38144, 38145, 38146, 39478, 39570, 40823, 41017, 41020, 42824, 45073, 45782, 46529, 47337, 47684, 58196, 73522, 93353, 93736, 98185, 99793, 100107, 100397, 100509, 100740, 102170, 102209, 102301, 102341, 102395, 102400, 102449, 102713, 102871, 102888, 102952, 103318, 103321, 103612, 103631, 103813, 103944, 103945, 103952, 103955, 103998, 105420, 105674, 106805, 107167 |
| Bronchiectasis | 2195, 15693, 20364, 32679, 41491, 56427, 109816 |
| Chronic Obstructive Pulmonary Disease | 794, 998, 1001, 5710, 9520, 9876, 10802, 10863, 10980, 11287, 12166, 14798, 16410, 18476, 18621, 18792, 23492, 26018, 26306, 28755, 33450, 34202, 34215, 37247, 37371, 38074, 42258, 42313, 45770, 45771, 45777, 45998, 46578, 60188, 65733, 67040, 93568, 104608, 104710, 104985, 105457, 106637 |
| Cystic Fibrosis | 100610, 36622, 69017, 18905, 93380, 65344, 100430, 106432, 73065, 102922, 18914, 103224, 6220, 49770, 100520 |
| Interstitial Lung Disease | 109815, 104915, 8317, 6051, 103472, 65060, 7791, 103753, 22536, 47782, 22835, 64799, 4910, 103475, 103785, 58841, 33980, 3859, 51410, 8303, 37365, 62233, 71853, 53095, 55552, 62442, 46977, 11833, 106515, 5519, 6837, 28229, 28853 |

**Supplementary Table 2: Code lists used for disease identification in CPRD.** CPRD = Clinical Practice Research Datalink.

|  | **Strict NTMD Cohort**  (n = 1,088)* | | | Males (n = 560) | | | Females (n = 528) | | | 18-34 years (n = 207) | | | 35-54 years (n = 297) | | | 55-74 years (n = 412) | | | 75+ years (n = 172) | | |
| --- | --- | --- | --- | --- | --- | --- | --- | --- | --- | --- | --- | --- | --- | --- | --- | --- | --- | --- | --- | --- | --- |
| Year |  |  |  |  |  |  |  |  |  |  |  |  |  |  |  |  |  |  |  |  |  |
|  | IR | LCL | UCL | IR | LCL | UCL | IR | LCL | UCL | IR | LCL | UCL | IR | LCL | UCL | IR | LCL | UCL | IR | LCL | UCL |
| 2006 | 3.85 | 3.28 | 4.51 | 4.06 | 3.25 | 5.07 | 3.64 | 2.89 | 4.58 | 3.31 | 2.35 | 4.66 | 3.30 | 2.49 | 4.38 | 4.43 | 3.32 | 5.91 | 5.63 | 3.74 | 8.47 |
| 2007 | 3.27 | 2.75 | 3.89 | 3.65 | 2.89 | 4.61 | 2.90 | 2.24 | 3.75 | 2.77 | 1.91 | 4.02 | 2.24 | 1.59 | 3.16 | 5.04 | 3.85 | 6.60 | 3.62 | 2.18 | 6.01 |
| 2008 | 2.86 | 2.38 | 3.44 | 2.72 | 2.08 | 3.56 | 2.99 | 2.32 | 3.86 | 2.76 | 1.91 | 4.00 | 2.52 | 1.83 | 3.48 | 3.13 | 2.22 | 4.40 | 3.62 | 2.18 | 6.01 |
| 2009 | 3.57 | 3.03 | 4.22 | 3.70 | 2.94 | 4.67 | 3.45 | 2.72 | 4.37 | 2.06 | 1.35 | 3.16 | 2.60 | 1.89 | 3.58 | 5.12 | 3.92 | 6.68 | 6.77 | 4.67 | 9.80 |
| 2010 | 2.98 | 2.48 | 3.57 | 2.78 | 2.17 | 3.58 | 3.11 | 2.38 | 4.04 | 1.98 | 1.28 | 3.07 | 2.23 | 1.58 | 3.16 | 4.42 | 3.31 | 5.90 | 4.40 | 2.77 | 6.98 |
| 2011 | 3.05 | 2.55 | 3.66 | 3.11 | 2.40 | 4.02 | 3.00 | 2.32 | 3.89 | 2.52 | 1.71 | 3.74 | 2.81 | 2.05 | 3.85 | 3.54 | 2.55 | 4.90 | 3.98 | 2.44 | 6.49 |
| 2012 | 2.43 | 1.98 | 2.99 | 2.56 | 1.92 | 3.40 | 2.31 | 1.72 | 3.10 | 1.52 | 0.92 | 2.52 | 1.48 | 0.95 | 2.29 | 4.18 | 3.09 | 5.65 | 3.52 | 2.08 | 5.94 |
| 2013 | 2.15 | 1.72 | 2.69 | 2.22 | 1.62 | 3.04 | 2.08 | 1.51 | 2.86 | 1.80 | 1.12 | 2.89 | 1.09 | 0.64 | 1.84 | 3.61 | 2.60 | 5.03 | 2.87 | 1.59 | 5.19 |
| 2014 | 2.45 | 1.97 | 3.05 | 2.50 | 1.83 | 3.40 | 2.40 | 1.76 | 3.28 | 1.17 | 0.63 | 2.18 | 1.37 | 0.84 | 2.24 | 3.93 | 2.82 | 5.47 | 5.36 | 3.42 | 8.41 |
| 2015 | 1.68 | 1.27 | 2.24 | 1.83 | 1.23 | 2.70 | 1.55 | 1.02 | 2.35 | 0.69 | 0.29 | 1.66 | 1.11 | 0.61 | 2.00 | 2.72 | 1.77 | 4.17 | 3.31 | 1.78 | 6.15 |
| 2016 | 1.28 | 0.88 | 1.85 | 1.49 | 0.91 | 2.43 | 1.08 | 0.61 | 1.89 | 0.89 | 0.37 | 2.14 | 1.15 | 0.60 | 2.21 | 1.79 | 0.99 | 3.24 | 1.28 | 0.41 | 3.97 |
|  | **Expanded NTMD Cohort**  (n = 10,327)* | | | Males (n = 4,523) | | | Females (n = 5,551) | | | 18-34 years (n = 1,748) | | | 35-54 years (n = 2,871) | | | 55-74 years (n = 3,814) | | | 75+ years (n = 894) | | |
| Year |  |  |  |  |  |  |  |  |  |  |  |  |  |  |  |  |  |  |  |  |  |
|  | IR | LCL | UCL | IR | LCL | UCL | IR | LCL | UCL | IR | LCL | UCL | IR | LCL | UCL | IR | LCL | UCL | IR | LCL | UCL |
| 2006 | 22.9 | 21.5 | 24.5 | 22.6 | 20.5 | 24.8 | 23.3 | 21.2 | 25.5 | 12.4 | 10.4 | 14.8 | 16.6 | 14.7 | 18.9 | 32.8 | 29.5 | 36.5 | 45.7 | 39.6 | 52.8 |
| 2007 | 22.8 | 21.3 | 24.3 | 21.4 | 19.4 | 23.6 | 24.1 | 22.0 | 26.3 | 13.9 | 11.8 | 16.4 | 15.4 | 13.6 | 17.6 | 35.6 | 32.2 | 39.4 | 37.9 | 32.4 | 44.3 |
| 2008 | 22.1 | 20.7 | 23.7 | 19.1 | 17.2 | 21.1 | 25.1 | 23.0 | 27.4 | 16.5 | 14.2 | 19.2 | 17.6 | 15.6 | 19.9 | 25.8 | 22.9 | 29.0 | 42.8 | 36.9 | 49.6 |
| 2009 | 22.3 | 20.9 | 23.8 | 18.8 | 17.0 | 20.8 | 25.7 | 23.6 | 28.1 | 15.0 | 12.8 | 17.6 | 17.8 | 15.8 | 20.1 | 31.1 | 27.9 | 34.7 | 33.6 | 28.5 | 39.7 |
| 2010 | 20.6 | 19.2 | 22.1 | 18.4 | 16.6 | 20.4 | 22.8 | 20.7 | 25.0 | 13.7 | 11.6 | 16.1 | 16.6 | 14.6 | 18.8 | 28.4 | 25.3 | 31.8 | 32.3 | 27.2 | 38.3 |
| 2011 | 23.4 | 21.9 | 25.0 | 22.1 | 20.0 | 24.3 | 24.7 | 22.6 | 27.0 | 17.7 | 15.2 | 20.5 | 20.3 | 18.0 | 22.8 | 27.5 | 24.5 | 31.0 | 37.8 | 32.3 | 44.3 |
| 2012 | 28.4 | 26.7 | 30.2 | 26.4 | 24.2 | 28.9 | 30.3 | 27.9 | 32.9 | 19.5 | 16.9 | 22.4 | 21.7 | 19.3 | 24.3 | 38.7 | 35.1 | 42.8 | 47.3 | 41.0 | 54.6 |
| 2013 | 30.8 | 29.0 | 32.6 | 28.6 | 26.2 | 31.2 | 32.8 | 30.3 | 35.6 | 22.2 | 19.4 | 25.4 | 23.0 | 20.5 | 25.8 | 40.6 | 36.8 | 44.9 | 52.8 | 46.0 | 60.6 |
| 2014 | 30.4 | 28.6 | 32.4 | 27.7 | 25.3 | 30.4 | 33.0 | 30.4 | 35.9 | 17.8 | 15.2 | 20.9 | 22.9 | 20.3 | 25.8 | 43.6 | 39.5 | 48.2 | 52.6 | 45.6 | 60.8 |
| 2015 | 37.4 | 35.2 | 39.7 | 35.1 | 32.1 | 38.4 | 39.5 | 36.4 | 43.0 | 23.0 | 19.7 | 26.8 | 27.4 | 24.3 | 30.9 | 51.8 | 47.0 | 57.2 | 67.7 | 59.0 | 77.6 |
| 2016 | 40.9 | 38.3 | 43.7 | 36.2 | 32.8 | 40.0 | 45.4 | 41.6 | 49.6 | 23.4 | 19.7 | 27.8 | 30.5 | 26.9 | 34.6 | 58.0 | 52.3 | 64.4 | 72.7 | 62.6 | 84.5 |

**Supplementary Table 3: Incidence of NTMD per 100,000 person-years overall, by sex, and by 20-year age groups.** Strict NTMD cohort on top. Expanded NTMD cohort on bottom. NTMD = nontuberculous mycobacterial disease. IR = incidence rate per 100,000 person-years. LCL = lower 95% confidence limit. UCL = upper 95% confidence limit. *174 patients and 216 patients in the strict and expanded NTMD cohorts, respectively, had prevalent disease at the start of follow-up and were therefore not included in these incidence calculations. **Notes:** Rates in tables provided are coloured using the scale below.

**Highest Values 50^th^ Percentile Lowest Values**


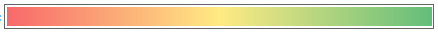


| **Strict NTMD Cohort** | **Males** (n = 560)* | | | | | | | | | | | | **Females** (n = 528)* | | | | | | | | | | | |
| --- | --- | --- | --- | --- | --- | --- | --- | --- | --- | --- | --- | --- | --- | --- | --- | --- | --- | --- | --- | --- | --- | --- | --- | --- |
|  | 18-34 years (n = 90) | | | 35-54 years (n = 172) | | | 55-74 years (n = 215) | | | 75+ years (n = 83) | | | 18-34 years (n = 117) | | | 35-54 years (n = 125) | | | 55-74 years (n = 197) | | | 75+ years (n = 89) | | |
| Year |  |  |  |  |  |  |  |  |  |  |  |  |  |  |  |  |  |  |  |  |  |  |  |  |
|  | IR | LCL | UCL | IR | LCL | UCL | IR | LCL | UCL | IR | LCL | UCL | IR | LCL | UCL | IR | LCL | UCL | IR | LCL | UCL | IR | LCL | UCL |
| 2006 | 2.77 | 1.64 | 4.67 | 4.31 | 3.05 | 6.09 | 4.10 | 2.67 | 6.28 | 6.97 | 3.86 | 12.6 | 3.87 | 2.47 | 6.07 | 2.25 | 1.38 | 3.67 | 4.75 | 3.21 | 7.03 | 4.78 | 2.71 | 8.42 |
| 2007 | 2.73 | 1.62 | 4.62 | 2.00 | 1.20 | 3.31 | 6.36 | 4.52 | 8.94 | 5.58 | 2.90 | 10.7 | 2.81 | 1.67 | 4.75 | 2.50 | 1.58 | 3.97 | 3.76 | 2.43 | 5.83 | 2.37 | 1.07 | 5.28 |
| 2008 | 2.54 | 1.47 | 4.37 | 2.80 | 1.83 | 4.30 | 2.68 | 1.59 | 4.53 | 3.07 | 1.28 | 7.38 | 2.99 | 1.80 | 4.97 | 2.23 | 1.37 | 3.64 | 3.56 | 2.27 | 5.58 | 3.98 | 2.14 | 7.40 |
| 2009 | 1.36 | 0.65 | 2.86 | 3.09 | 2.05 | 4.64 | 5.38 | 3.71 | 7.79 | 8.52 | 5.04 | 14.4 | 2.78 | 1.65 | 4.69 | 2.10 | 1.27 | 3.49 | 4.87 | 3.31 | 7.15 | 5.62 | 3.33 | 9.48 |
| 2010 | 1.37 | 0.65 | 2.88 | 3.01 | 1.98 | 4.57 | 4.67 | 3.13 | 6.97 | 4.88 | 2.44 | 9.76 | 2.59 | 1.51 | 4.47 | 1.43 | 0.77 | 2.65 | 4.18 | 2.75 | 6.34 | 4.07 | 2.19 | 7.57 |
| 2011 | 1.61 | 0.80 | 3.21 | 3.26 | 2.17 | 4.90 | 3.59 | 2.26 | 5.70 | 5.54 | 2.88 | 10.6 | 3.45 | 2.15 | 5.55 | 2.35 | 1.44 | 3.83 | 3.48 | 2.20 | 5.53 | 2.92 | 1.52 | 5.61 |
| 2012 | 1.21 | 0.55 | 2.70 | 1.16 | 0.58 | 2.33 | 5.05 | 3.41 | 7.48 | 4.93 | 2.46 | 9.86 | 1.83 | 0.95 | 3.51 | 1.80 | 1.02 | 3.17 | 3.33 | 2.07 | 5.35 | 2.54 | 1.14 | 5.66 |
| 2013 | 2.12 | 1.14 | 3.95 | 0.61 | 0.23 | 1.63 | 4.20 | 2.71 | 6.51 | 3.17 | 1.32 | 7.62 | 1.48 | 0.70 | 3.10 | 1.58 | 0.85 | 2.93 | 3.05 | 1.84 | 5.06 | 2.66 | 1.20 | 5.92 |
| 2014 | 1.41 | 0.63 | 3.14 | 1.52 | 0.79 | 2.92 | 3.87 | 2.41 | 6.23 | 5.45 | 2.73 | 10.9 | 0.93 | 0.35 | 2.49 | 1.21 | 0.58 | 2.54 | 3.98 | 2.51 | 6.32 | 5.30 | 2.94 | 9.57 |
| 2015 | 0.83 | 0.27 | 2.58 | 1.79 | 0.93 | 3.45 | 2.37 | 1.23 | 4.55 | 3.17 | 1.19 | 8.46 | 0.55 | 0.14 | 2.21 | 0.41 | 0.10 | 1.63 | 3.07 | 1.74 | 5.40 | 3.41 | 1.53 | 7.58 |
| 2016 | 0.72 | 0.18 | 2.86 | 1.52 | 0.68 | 3.39 | 1.99 | 0.89 | 4.42 | 2.04 | 0.51 | 8.14 | 1.07 | 0.34 | 3.31 | 0.77 | 0.25 | 2.40 | 1.61 | 0.67 | 3.86 | 0.73 | 0.10 | 5.21 |
| **Expanded NTMD Cohort** | **Males** (n = 4,649)* | | | | | | | | | | | | **Females** (n = 5,678)* | | | | | | | | | | | |
|  | 18-34 years (n = 629) | | | 35-54 years (n = 1,233) | | | 55-74 years (n = 1,845) | | | 75+ years (n = 942) | | | 18-34 years (n = 1,119) | | | 35-54 years (n = 1,638) | | | 55-74 years (n = 1,969) | | | 75+ years (n = 952) | | |
| Year |  |  |  |  |  |  |  |  |  |  |  |  |  |  |  |  |  |  |  |  |  |  |  |  |
|  | IR | LCL | UCL | IR | LCL | UCL | IR | LCL | UCL | IR | LCL | UCL | IR | LCL | UCL | IR | LCL | UCL | IR | LCL | UCL | IR | LCL | UCL |
| 2006 | 10.3 | 7.83 | 13.5 | 16.7 | 14.0 | 19.9 | 32.3 | 27.7 | 37.6 | 58.3 | 47.5 | 71.5 | 14.7 | 11.6 | 18.5 | 16.6 | 13.8 | 19.8 | 33.4 | 28.8 | 38.8 | 37.9 | 31.0 | 46.3 |
| 2007 | 11.5 | 8.93 | 14.9 | 14.4 | 11.9 | 17.4 | 34.9 | 30.2 | 40.3 | 42.2 | 33.3 | 53.5 | 16.3 | 13.1 | 20.2 | 16.5 | 13.8 | 19.8 | 36.3 | 31.5 | 41.8 | 35.2 | 28.6 | 43.3 |
| 2008 | 12.1 | 9.43 | 15.5 | 14.0 | 11.6 | 17.0 | 22.6 | 18.9 | 27.1 | 52.9 | 42.8 | 65.3 | 21.0 | 17.3 | 25.4 | 21.3 | 18.2 | 25.0 | 28.9 | 24.6 | 33.8 | 36.2 | 29.5 | 44.5 |
| 2009 | 9.74 | 7.38 | 12.8 | 12.9 | 10.6 | 15.7 | 28.6 | 24.4 | 33.6 | 42.6 | 33.7 | 53.9 | 20.4 | 16.9 | 24.8 | 23.0 | 19.7 | 26.8 | 33.5 | 29.0 | 38.8 | 27.7 | 21.9 | 35.1 |
| 2010 | 7.26 | 5.26 | 10.0 | 14.1 | 11.6 | 17.1 | 28.4 | 24.2 | 33.5 | 40.9 | 32.2 | 52.0 | 20.2 | 16.6 | 24.5 | 19.1 | 16.1 | 22.6 | 28.3 | 24.1 | 33.2 | 26.5 | 20.8 | 33.8 |
| 2011 | 12.5 | 9.71 | 16.0 | 16.7 | 14.0 | 20.0 | 31.5 | 27.0 | 36.9 | 45.6 | 36.3 | 57.3 | 23.0 | 19.1 | 27.6 | 23.9 | 20.5 | 27.9 | 23.6 | 19.8 | 28.2 | 32.5 | 26.1 | 40.6 |
| 2012 | 13.2 | 10.3 | 16.8 | 17.8 | 14.9 | 21.2 | 39.3 | 34.1 | 45.2 | 64.2 | 53.0 | 77.8 | 25.8 | 21.7 | 30.7 | 25.7 | 22.1 | 29.9 | 38.2 | 33.2 | 44.0 | 35.7 | 28.8 | 44.2 |
| 2013 | 18.1 | 14.6 | 22.3 | 17.8 | 14.8 | 21.4 | 41.4 | 36.0 | 47.6 | 66.1 | 54.6 | 80.1 | 26.4 | 22.1 | 31.4 | 28.4 | 24.5 | 32.8 | 39.9 | 34.7 | 45.9 | 43.5 | 35.7 | 53.1 |
| 2014 | 11.8 | 8.91 | 15.5 | 18.8 | 15.6 | 22.6 | 43.6 | 37.8 | 50.2 | 62.9 | 51.3 | 77.1 | 23.9 | 19.6 | 29.0 | 27.1 | 23.1 | 31.7 | 43.7 | 38.0 | 50.2 | 45.4 | 37.1 | 55.6 |
| 2015 | 16.1 | 12.5 | 20.8 | 26.9 | 22.7 | 31.9 | 46.6 | 40.2 | 54.0 | 87.6 | 72.6 | 106 | 29.8 | 24.7 | 36.0 | 27.9 | 23.6 | 33.0 | 56.9 | 49.8 | 64.8 | 53.5 | 43.7 | 65.5 |
| 2016 | 17.5 | 13.3 | 23.2 | 24.1 | 19.7 | 29.5 | 56.0 | 48.2 | 65.2 | 76.7 | 61.1 | 96.1 | 29.2 | 23.5 | 36.3 | 37.0 | 31.4 | 43.6 | 59.9 | 51.9 | 69.2 | 69.9 | 57.2 | 85.5 |

**Supplementary Table 4: Incidence of NTMD per 100,000 person-years by sex and 20-year age groups.** Strict NTMD cohort on top. Expanded NTMD cohort on bottom. NTMD = nontuberculous mycobacterial disease. IR = incidence rate per 100,000 person-years. LCL = lower 95% confidence limit. UCL = upper 95% confidence limit. *174 patients and 216 patients in the strict and expanded NTMD cohorts, respectively, had prevalent disease at the start of follow-up and were therefore not included in these incidence calculations. **Notes:** Rates in tables provided are coloured using the scale below.

**Highest Values 50^th^ Percentile Lowest Values**


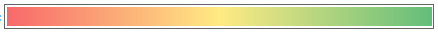


|  | **Strict CRD-NTMD Cohort**  (n = 417)* | | | **Expanded CRD-NTMD Cohort**  (n = 4,062)* | | |
| --- | --- | --- | --- | --- | --- | --- |
| Year |  |  |  |  |  |  |
|  | IR | LCL | UCL | IR | LCL | UCL |
| 2006 | 12.5 | 9.39 | 16.5 | 90.6 | 81.6 | 100.6 |
| 2007 | 13.2 | 10.1 | 17.4 | 91.0 | 82.0 | 100.9 |
| 2008 | 11.3 | 8.29 | 15.5 | 74.3 | 66.2 | 83.3 |
| 2009 | 16.4 | 12.4 | 21.6 | 83.2 | 74.7 | 92.8 |
| 2010 | 13.2 | 9.58 | 18.3 | 75.2 | 67.1 | 84.4 |
| 2011 | 12.4 | 9.28 | 16.4 | 86.8 | 77.9 | 96.7 |
| 2012 | 10.9 | 7.87 | 15.0 | 106.8 | 96.8 | 117.8 |
| 2013 | 8.87 | 6.28 | 12.6 | 117.5 | 106.8 | 129.3 |
| 2014 | 13.9 | 10.3 | 18.8 | 132.2 | 120.4 | 145.3 |
| 2015 | 11.3 | 7.53 | 17.1 | 168.3 | 153.8 | 184.1 |
| 2016 | 7.40 | 3.98 | 13.8 | 175.2 | 158.4 | 193.7 |

**Supplementary Table 5: Incidence of NTMD per 100,000 person-years in patients with underlying respiratory disease overall, by sex, and by 20-year age groups.** Strict NTMD cohort with underlying respiratory disease on left. Expanded NTMD cohort with underlying respiratory disease on right. CRD = chronic respiratory disease. NTMD = nontuberculous mycobacterial disease. IR = incidence rate per 100,000 person-years. LCL = lower 95% confidence limit. UCL = upper 95% confidence limit. *67 patients and 83 patients in the strict and expanded NTMD cohorts, respectively, had prevalent disease at the start of follow-up and were therefore not included in these incidence calculations. **Notes:** Rates in tables provided are coloured using the scale below. In this table, rates are coloured relative only to the other annual rates in the same cohort.

**Highest Values 50^th^ Percentile Lowest Values**


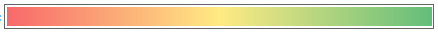


|  | **Strict NTMD Cohort**  (n = 1,262) | | | Males (n = 661) | | | Females (n = 601) | | | 18-34 years (n = 234) | | | 35-54 years (n = 340) | | | 55-74 years (n = 482) | | | 75+ years (n = 206) | | |
| --- | --- | --- | --- | --- | --- | --- | --- | --- | --- | --- | --- | --- | --- | --- | --- | --- | --- | --- | --- | --- | --- |
| Year |  |  |  |  |  |  |  |  |  |  |  |  |  |  |  |  |  |  |  |  |  |
|  | PR | LCL | UCL | PR | LCL | UCL | PR | LCL | UCL | PR | LCL | UCL | PR | LCL | UCL | PR | LCL | UCL | PR | LCL | UCL |
| 2006 | 7.68 | 7.68 | 7.68 | 8.31 | 8.31 | 8.31 | 7.06 | 7.06 | 7.06 | 4.62 | 4.62 | 4.62 | 6.22 | 6.22 | 6.22 | 10.1 | 10.1 | 10.1 | 14.5 | 14.5 | 14.5 |
| 2007 | 7.06 | 7.06 | 7.06 | 7.55 | 7.55 | 7.55 | 6.59 | 6.59 | 6.59 | 4.56 | 4.56 | 4.56 | 4.58 | 4.58 | 4.58 | 10.6 | 10.6 | 10.6 | 13.1 | 13.1 | 13.1 |
| 2008 | 6.34 | 6.34 | 6.34 | 7.09 | 7.09 | 7.09 | 5.60 | 5.6 | 5.6 | 4.23 | 4.23 | 4.23 | 4.98 | 4.98 | 4.98 | 8.90 | 8.90 | 8.90 | 9.91 | 9.91 | 9.91 |
| 2009 | 6.86 | 6.86 | 6.86 | 7.19 | 7.19 | 7.19 | 6.55 | 6.55 | 6.55 | 3.82 | 3.82 | 3.82 | 5.20 | 5.20 | 5.20 | 10.0 | 10.0 | 10.0 | 12.3 | 12.3 | 12.3 |
| 2010 | 7.14 | 7.14 | 7.14 | 7.42 | 7.42 | 7.42 | 6.87 | 6.87 | 6.87 | 4.13 | 4.13 | 4.13 | 5.15 | 5.15 | 5.15 | 11.3 | 11.3 | 11.3 | 11.2 | 11.2 | 11.2 |
| 2011 | 6.86 | 6.86 | 6.86 | 6.81 | 6.81 | 6.81 | 6.90 | 6.9 | 6.9 | 4.89 | 4.89 | 4.89 | 4.87 | 4.87 | 4.87 | 9.65 | 9.65 | 9.65 | 11.6 | 11.6 | 11.6 |
| 2012 | 6.27 | 6.27 | 6.27 | 6.24 | 6.24 | 6.24 | 6.29 | 6.29 | 6.29 | 3.94 | 3.94 | 3.94 | 4.13 | 4.13 | 4.13 | 9.41 | 9.41 | 9.41 | 11.5 | 11.5 | 11.5 |
| 2013 | 5.20 | 5.20 | 5.20 | 4.99 | 4.99 | 4.99 | 5.40 | 5.4 | 5.4 | 2.96 | 2.96 | 2.96 | 3.47 | 3.47 | 3.47 | 8.62 | 8.62 | 8.62 | 8.05 | 8.05 | 8.05 |
| 2014 | 5.66 | 5.66 | 5.66 | 5.68 | 5.68 | 5.68 | 5.65 | 5.65 | 5.65 | 3.06 | 3.06 | 3.06 | 3.24 | 3.24 | 3.24 | 9.07 | 9.07 | 9.07 | 11.6 | 11.6 | 11.6 |
| 2015 | 5.17 | 5.17 | 5.17 | 4.80 | 4.80 | 4.80 | 5.52 | 5.52 | 5.52 | 2.12 | 2.12 | 2.12 | 3.58 | 3.58 | 3.58 | 7.32 | 7.32 | 7.32 | 12.4 | 12.4 | 12.4 |
| 2016 | 4.70 | 4.70 | 4.70 | 4.61 | 4.61 | 4.61 | 4.79 | 4.79 | 4.79 | 2.42 | 2.42 | 2.42 | 2.94 | 2.94 | 2.94 | 6.99 | 6.99 | 6.99 | 10.2 | 10.2 | 10.2 |

**Supplementary Table 6: Prevalence of NTMD per 100,000 population overall, by sex, and by 20-year age groups.** NTMD = nontuberculous mycobacterial disease. PR = prevalence rate per 100,000 population. LCL = lower 95% confidence limit. UCL = upper 95% confidence limit. **Notes:** Rates in tables provided are coloured using the scale below.

**Highest Values 50^th^ Percentile Lowest Values**


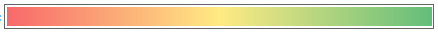


| **Strict NTMD Cohort** | **Males** (n = 661) | | | | | | | | | | | | **Females** (n = 601) | | | | | | | | | | | |
| --- | --- | --- | --- | --- | --- | --- | --- | --- | --- | --- | --- | --- | --- | --- | --- | --- | --- | --- | --- | --- | --- | --- | --- | --- |
|  | 18-34 years (n = 103) | | | 35-54 years (n = 199) | | | 55-74 years (n = 259) | | | 75+ years (n = 100) | | | 18-34 years (n = 131) | | | 35-54 years (n = 141) | | | 55-74 years (n = 223) | | | 75+ years (n = 106) | | |
| Year |  |  |  |  |  |  |  |  |  |  |  |  |  |  |  |  |  |  |  |  |  |  |  |  |
|  | PR | LCL | UCL | PR | LCL | UCL | PR | LCL | UCL | PR | LCL | UCL | PR | LCL | UCL | PR | LCL | UCL | PR | LCL | UCL | PR | LCL | UCL |
| 2006 | 3.67 | 3.67 | 3.67 | 7.57 | 7.57 | 7.57 | 11.2 | 11.2 | 11.2 | 17.9 | 17.9 | 17.9 | 5.60 | 5.60 | 5.60 | 4.80 | 4.80 | 4.80 | 8.96 | 8.96 | 8.96 | 12.4 | 12.4 | 12.4 |
| 2007 | 4.58 | 4.58 | 4.58 | 4.55 | 4.55 | 4.55 | 11.8 | 11.8 | 11.8 | 17.5 | 17.5 | 17.5 | 4.53 | 4.53 | 4.53 | 4.61 | 4.61 | 4.61 | 9.46 | 9.46 | 9.46 | 10.4 | 10.4 | 10.4 |
| 2008 | 4.18 | 4.18 | 4.18 | 5.34 | 5.34 | 5.34 | 10.3 | 10.4 | 10.4 | 14.1 | 14.1 | 14.1 | 4.28 | 4.28 | 4.28 | 4.60 | 4.60 | 4.60 | 7.49 | 7.49 | 7.49 | 7.17 | 7.17 | 7.17 |
| 2009 | 2.84 | 2.84 | 2.84 | 5.89 | 5.89 | 5.89 | 10.4 | 10.4 | 10.4 | 17.0 | 17.0 | 17.0 | 4.83 | 4.83 | 4.83 | 4.48 | 4.48 | 4.48 | 9.72 | 9.72 | 9.72 | 9.21 | 9.21 | 9.21 |
| 2010 | 2.09 | 2.09 | 2.09 | 6.40 | 6.4 | 6.4 | 11.6 | 11.6 | 11.6 | 15.8 | 15.8 | 15.8 | 6.21 | 6.21 | 6.21 | 3.83 | 3.83 | 3.83 | 11.0 | 11.0 | 11.0 | 8.12 | 8.12 | 8.12 |
| 2011 | 2.72 | 2.72 | 2.72 | 5.63 | 5.63 | 5.63 | 9.89 | 9.89 | 9.89 | 15.3 | 15.3 | 15.3 | 7.09 | 7.09 | 7.09 | 4.08 | 4.08 | 4.08 | 9.41 | 9.41 | 9.41 | 9.11 | 9.11 | 9.11 |
| 2012 | 2.35 | 2.35 | 2.35 | 3.77 | 3.77 | 3.77 | 11.1 | 11.1 | 11.1 | 14.1 | 14.1 | 14.1 | 5.53 | 5.53 | 5.53 | 4.49 | 4.49 | 4.49 | 7.80 | 7.80 | 7.80 | 9.72 | 9.72 | 9.72 |
| 2013 | 2.67 | 2.67 | 2.67 | 2.43 | 2.43 | 2.43 | 9.39 | 9.39 | 9.39 | 9.48 | 9.48 | 9.48 | 3.26 | 3.26 | 3.26 | 4.53 | 4.53 | 4.53 | 7.87 | 7.87 | 7.87 | 7.06 | 7.06 | 7.06 |
| 2014 | 2.95 | 2.95 | 2.95 | 2.86 | 2.86 | 2.86 | 10.0 | 10.0 | 10.0 | 12.3 | 12.3 | 12.3 | 3.17 | 3.17 | 3.17 | 3.63 | 3.63 | 3.63 | 8.17 | 8.17 | 8.17 | 11.1 | 11.1 | 11.1 |
| 2015 | 1.86 | 1.86 | 1.86 | 3.94 | 3.94 | 3.94 | 6.77 | 6.77 | 6.77 | 11.0 | 11.0 | 11.0 | 2.38 | 2.38 | 2.38 | 3.22 | 3.22 | 3.22 | 7.84 | 7.84 | 7.84 | 13.5 | 13.5 | 13.5 |
| 2016 | 1.73 | 1.73 | 1.73 | 3.04 | 3.04 | 3.04 | 7.27 | 7.27 | 7.27 | 11.2 | 11.2 | 11.2 | 3.10 | 3.10 | 3.10 | 2.84 | 2.84 | 2.84 | 6.73 | 6.73 | 6.73 | 9.52 | 9.52 | 9.52 |

**Supplementary Table 7: Prevalence of NTMD per 100,000 population by sex and 20-year age groups.** NTMD = nontuberculous mycobacterial disease. PR = prevalence per 100,000 population. LCL = lower 95% confidence limit. UCL = upper 95% confidence limit. **Notes:** Rates in tables provided are coloured using the scale below.

**Highest Values 50^th^ Percentile Lowest Values**


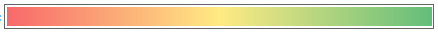


|  | **Strict CRD-NTMD Cohort**  (n = 482) | | |
| --- | --- | --- | --- |
| Year |  |  |  |
|  | PR | LCL | UCL |
| 2006 | 27.4 | 27.4 | 27.4 |
| 2007 | 29.2 | 29.2 | 29.2 |
| 2008 | 27.3 | 27.3 | 27.3 |
| 2009 | 29.8 | 29.8 | 29.8 |
| 2010 | 27.8 | 27.8 | 27.8 |
| 2011 | 28.5 | 28.5 | 28.5 |
| 2012 | 28.0 | 28.0 | 28.0 |
| 2013 | 24.3 | 24.3 | 24.3 |
| 2014 | 29.0 | 29.0 | 29.0 |
| 2015 | 26.0 | 26.0 | 26.0 |
| 2016 | 26.8 | 26.8 | 26.8 |

**Supplementary Table 8: Prevalence of NTMD in patients with underlying respiratory disease per 100,000 population overall.** CRD = chronic respiratory disease. NTMD = nontuberculous mycobacterial disease. PR = prevalence per 100,000 population. LCL = lower 95% confidence limit. UCL = upper 95% confidence limit. **Notes:** Rates in tables provided are coloured using the scale below.

**Highest Values 50^th^ Percentile Lowest Values**


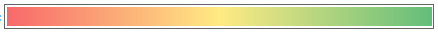

Supplement: Supplementary file 1 — (DOCX 66 kb) [file 10096_2018_3315_MOESM1_ESM.docx]
